# The Association Between Anemia and Skin Autofluorescence, a Marker for Advanced Glycation End Product Accumulation

**DOI:** 10.1097/HS9.0000000000000470

**Published:** 2020-10-26

**Authors:** Hanneke J.C.M. Wouters, Melanie M. van der Klauw, Bruce H.R. Wolffenbuttel, Gerwin Huls, Isabelle A. van Zeventer

**Affiliations:** 1 Department of Hematology, University of Groningen, University Medical Center Groningen, Groningen, The Netherlands; 2 Department of Endocrinology, University of Groningen, University Medical Center Groningen, Groningen, The Netherlands.

Advanced glycation end products (AGEs) are formed by glycation and oxidation of proteins and other molecules.^1,2^ Tissue AGE accumulation may be assessed by the non-invasive method of skin autofluorescence (SAF).^2^ AGE levels have previously been linked to diabetes, diabetic (micro)vascular complications and cardiovascular morbidity and mortality.^1,3^

Accumulation of AGEs increases with advancing age. It has been suggested that AGEs contribute to a multi system decline with aging, including anemia.^4^ As one of the major hallmarks of hematopoietic aging, anemia is associated with significant morbidity and mortality. Despite increasing diagnostic possibilities, the underlying pathophysiology is currently incompletely understood. A substantial proportion of anemic cases in the elderly remains unexplained.^5^ Although AGEs have been studied extensively in the context of other age-related conditions, little is known about the relationship between AGEs and hematopoietic aging phenotypes. Few studies have reported on an association between AGEs and anemia in small cohorts of community-dwelling individuals and diabetic patients.^6–8^ However, data from large cohorts of aging individuals, evaluating the relation between AGEs and anemia, are lacking.

We studied SAF levels and their relation to (different subtypes of) anemia in the Lifelines cohort, a multi-disciplinary prospective population-based cohort study examining the health and health-related behaviors of 167,729 persons living in the North of The Netherlands. The local ethics committee approved the research protocol and informed consent was obtained from every participant.^9^ For the present study, we included all subjects (aged 18–92 years) of Western European descent of whom hemoglobin concentration and SAF measurements without use of tanning agents were available (n = 78,917). Since SAF is strongly associated with diabetes, cardiovascular disease and impaired renal function,^3^ individuals with diabetes, self-reported history of cardiovascular disease and/or estimated glomerular filtration rate (eGFR; calculated with CKD-EPI equation) <30 ml/min/1.73 m^2^ were excluded, leaving a cohort of 74,603 for subsequent analyses. Follow-up hemoglobin concentrations were available for 54,304 participants after a median of 49 months (range 13–109). Anemia was defined according to WHO definitions: hemoglobin concentration <13.0 g/dL in males and <12.0 g/dL in females. Mild anemia was defined as a hemoglobin concentration between 11.0 and 12.99 g/dL in males and between 11.0 and 11.99 g/dL in females, whereas in both males and females moderate and severe anemia were defined as hemoglobin concentration between 10.0–10.99 g/dL and <10.0 g/dL respectively. SAF was measured using the non-invasive AGE reader (Diagnoptics, Groningen, The Netherlands), as described previously.^2^ For univariable analyses, SAF measurements were age- and sex-adjusted by calculating Z-scores. We evaluated between-group differences using one-way ANOVA, Kruskal-Wallis test, or Chi-square test, as appropriate. Linear regression analyses were performed to determine the association between hemoglobin concentration or anemia and SAF. Subsequently, logistic regression was used to assess the association between SAF at baseline and the development of anemia over time. Multivariable models were constructed correcting for known possible confounders: age, sex, eGFR, high-density lipoprotein cholesterol, glycated hemoglobin, triglycerides, body mass index, current smoking, pack years and number of cups of coffee per day.^3^ Standardized regression coefficients (β) and odds ratios (OR) with 95% confidence intervals (CI) are reported. Statistical analyses were performed using IBM SPSS software, version 23.0 (SPSS Inc., Chicago, IL). A p value <0.05 was considered statistically significant. Details of the Lifelines cohort regarding clinical examination, biochemical measurements and definition of anemia subtypes are described in the Supplemental Digital Content.

We included 74,603 participants with a mean age of 44.0 (± 12.3) years of whom 43,946 (58.9%) were females. Baseline characteristics according to the presence of anemia are shown in Supplemental Digital Content, Table 1. Anemia was detected in 4.2% (3,128/74,603) individuals. Mean baseline SAF Z-scores were 0.060 (standard deviation (SD) 0.98) for individuals with anemia vs −0.003 (SD 1.00) for individuals without anemia (p = 0.001) (Fig. 1A). Individuals with severe (mean 0.220, SD 0.98, n = 223) and moderate (mean 0.119, SD 0.97, n = 434) anemia had higher SAF Z-scores as compared to individuals with mild anemia (mean 0.036, SD 0.98, n = 2,471) or absence of anemia (mean −0.003, SD 1.00) (p < 0.001) (Fig. 1B). Since anemia can be considered as a consequence of aging of the hematopoietic system, we separately evaluated the associations for older individuals (≥60 years). Overall, the severity of anemia in older individuals (mean hemoglobin 12.3 ± 0.6 g/dL in n = 115 males and 11.5 ± 0.7 g/dL in n = 132 females) was comparable to those aged <60 years (mean hemoglobin 12.5 ± 0.5 g/dL in n = 246 males and 11.3 ± 0.8 g/dL in n = 2,635 females). Older individuals with anemia had relatively higher SAF Z-scores (mean 0.124, SD 0.97, n = 247) as compared to individuals <60 years with anemia (mean 0.055, SD 0.98, n = 2,881), although this difference was not significant (p = 0.29) (Fig. 1C).

**Figure 1:**
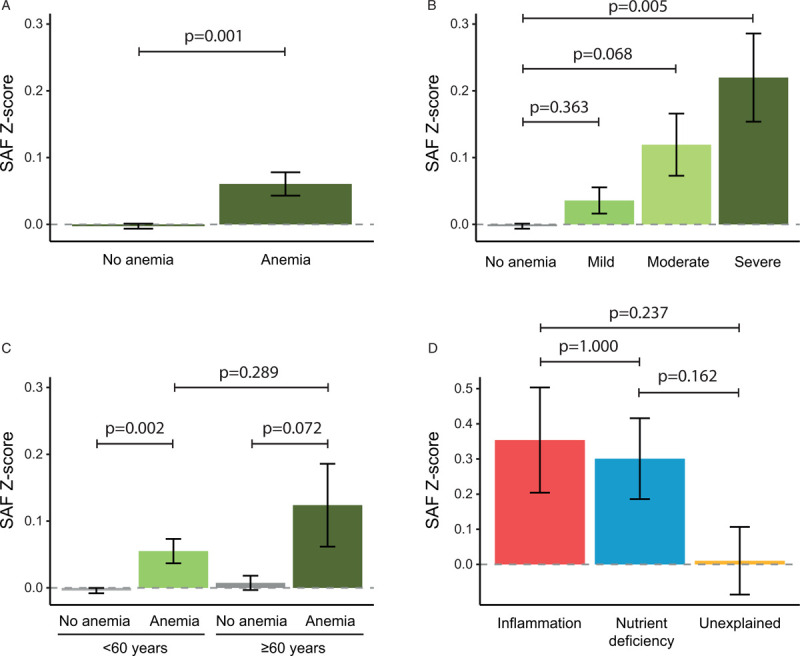
**SAF Z-scores according to presence of anemia (A), severity of anemia (B), age and presence of anemia (C) and the type of anemia (D).** Mild anemia was defined as a hemoglobin concentration between 11.0 and 12.99 g/dL in males and between 11.0 and 11.99 g/dL in females, whereas moderate and severe anemia were defined as hemoglobin concentration between 10.0 and 10.99 g/dL and <10.0 g/dL respectively. Data on the type of anemia were available for anemic individuals aged ≥60 years. Bars represent mean values, with error bars representing the standard error. SAF = skin autofluorescence.

Additional biochemical tests on stored plasma samples were performed to determine the type of anemia in anemic individuals ≥60 years (Supplemental Materials and Methods). In total, we were able to relate SAF levels to type of anemia in 211 individuals. Females with nutrient deficiency anemia had lower hemoglobin concentrations (mean 11.1 g/dL, SD 1.0, n = 38) than females with anemia of inflammation (mean 11.8 g/dL, SD 0.2, n = 16) or unexplained anemia (mean 11.7 g/dL, SD 0.3, n = 76), whereas hemoglobin concentrations in males were comparable between the subtypes of anemia. Overall, highest SAF Z-scores were detected for individuals with anemia of inflammation (mean 0.354, SD 0.85, n = 32) and nutrient deficiency anemia (mean 0.301, SD 0.94, n = 67) as compared to individuals with unexplained anemia (mean 0.010, SD 1.02, n = 112) (p = 0.072) (Fig. 1D).

Next, we performed multivariable linear regression analyses in a subset of individuals with available data (n = 70,634) to control for known possible confounders (Table 1). The presence of anemia positively associated with SAF (β = 0.021, p < 0.001). We also observed an inverse correlation between absolute hemoglobin concentration and SAF (β = −0.43, p < 0.001).

**Table 1:**
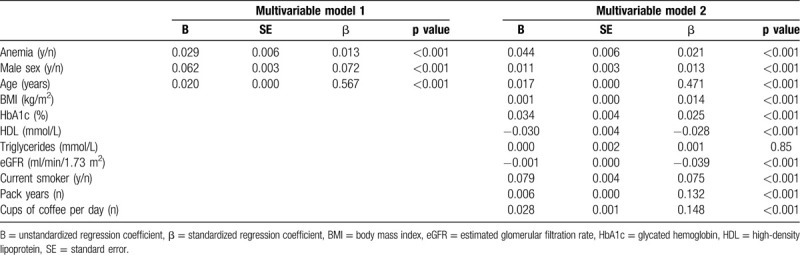
Multivariable Linear Regression for the Association Between Anemia and SAF.

Finally, we questioned whether SAF predicted for the development of anemia over time. Over a median period of 49 months, 1,296 evaluable individuals (2.5%) newly developed anemia. SAF was associated with development of anemia over time after correction for known confounders in the multivariable model (OR 1.4, 95% CI 1.1 - 1.6, p = 0.001).

This study presents the first large-scale assessment of SAF in the context of anemia, demonstrating that SAF and anemia are associated in community-dwelling individuals. Moreover, SAF predicted for development of anemia over time. These findings confirm previously reported associations between AGEs and anemia in population-based cohorts of 751^6^ and 1,036^7^ individuals and in a cohort of individuals with diabetes (n = 604).^8^

Several mechanisms may explain the relation between AGEs and anemia. First, AGEs are known to trigger pro-inflammatory signaling cascades^1,4^ that may be implicated in the anemia of inflammation. Higher levels of AGEs were indeed found in individuals with anemia of inflammation. Second, as mentioned previously,^6,7^ AGEs may contribute to membrane modifications and alterations in deformability of erythrocytes, thereby increasing the interaction with the endothelial surface.^10–12^ Whether this affects erythrocyte lifespan has not yet been proven. Third, reduced tissue oxygenation associated with anemia may contribute to low-level formation and accumulation of AGEs.^8^ Last, AGEs accumulate continuously over the life span of an individual.^4^ As anemia emerges with aging, these two phenomena may represent independent markers of higher biological age and increased vulnerability, rather than being causally related.

Several limitations of SAF measurements in the context of anemia should be addressed. As is understood for now, most AGEs are not fluorescent, making it impossible to draw conclusions about individual AGE compounds when using SAF for AGE determination. However, previous validation studies have reported strong correlations between SAF and both fluorescent (pentosidine) and non-fluorescent (Nε-carboxymethyl-lysine (CML) and Nε-carboxyethyl-lysine (CEL)) AGE levels in skin biopsies.^2,13^ Thus, SAF may be used as a marker of a broader AGE pool than fluorescent AGEs alone. Second, SAF measurements may be affected by chromophores and other molecules in the skin.^14^ Well-recognized confounders are skin pigmentation (eg, melanin) and tanning agents, which have been accounted for in this study by applying strict inclusion criteria. Although skin redness is regarded of less importance for variation in fluorescence,^15^ we cannot fully exclude minor variance by the amount of hemoglobin molecules in the skin. Nevertheless, the present cohort offered a valuable opportunity to study SAF levels as a proxy for AGE at large scale, thereby confirming previous associations between AGEs in plasma and anemia.

Given the value of AGEs as a biomarker for (co)morbid conditions and outcome and their potential modifiability,^1,4,16,17^ the demonstrated relation between SAF and anemia of aging deserves further attention.

## Acknowledgments

The authors wish to acknowledge the services of the Lifelines Cohort Study, the contributing research centers delivering data to Lifelines, and all the study participants. The authors would also like to thank all local investigators and operational team members of the MDS-RIGHT project for their contribution.

## Source of funding

The Lifelines Biobank initiative has been made possible by subsidy from the Dutch Ministry of Health, Welfare and Sport, the Dutch Ministry of Economic Affairs, the University Medical Center Groningen (UMCG the Netherlands), University Groningen and the Northern Provinces of the Netherlands. This work is part of the MDS-RIGHT project, which has received funding from the European Union's Horizon 2020 research and innovation program under grant agreement No 634789 - ’Providing the right care to the right patient with MyeloDysplastic Syndrome at the right time’.

## Supplementary Material

Supplemental Digital Content
