# Supplementary material for: The Association Between Anemia and Skin Autofluorescence, a Marker for Advanced Glycation End Product Accumulation
Source: Hemasphere. 2020 Oct 26;4(6):e470. doi: 10.1097/HS9.0000000000000470 (PMC7593064; doi:10.1097/HS9.0000000000000470)
Supplement: Supplemental Digital Content [file hs9-4-e470-s001.pdf]

Supplemental Digital Content (SDC)

SDC, Materials and Methods

Study design

Lifelines is a multi-disciplinary prospective population-based cohort study examining in a unique three-generation design the health and health-related behaviors of 167729 persons living in the North of The Netherlands. It employs a broad range of investigative procedures in assessing the biomedical, socio-demographic, behavioral, physical and psychological factors which contribute to the health and disease of the general population, with a special focus on multi-morbidity and complex genetics.[1,2]

Clinical parameters

All subjects completed a self-administered questionnaire on medical history, past and current diseases, use of medication, and health behavior at home. Medication use was verified by a certified research assistant, and scored by ATC code. Body weight was measured wearing light clothing without shoes to the nearest 0.1kg, whereas height was measured to the nearest 0.5cm. Body mass index was calculated as kg/m^2. Participants completed an extensive questionnaire which included structured questions about smoking behavior and coffee consumption. An individual was defined as being a current smoker if he/she answered positively to the question 'do you smoke now or have you been smoking in the last month?'. Pack-years of smoking were calculated as the number of packs of cigarettes (1 pack=20 cigarettes) smoked per day times the number of years of smoking. Coffee consumption was recorded as the number of cups of coffee per day. Presence of diabetes was defined based on either self-report, elevated fasting glucose (>7.0 mmol/L) or anti-diabetic drug use.[3] Cardiovascular disease was defined as present when participants reported history of myocardial infarction, percutaneous transluminal coronary angioplasty, coronary artery bypass grafting, cerebrovascular accident, or peripheral artery vascular surgery, as described previously.[4]

Skin autofluorescence (SAF)

SAF was measured using the non-invasive AGE reader (Diagnoptics, Groningen, The Netherlands). The AGE reader illuminates a skin surface of approximately 4 cm^2, guarded against surrounding light, with an excitation light source with wavelength between 300 and 420 nm (peak intensity at ~370 nm). Emission light and reflected excitation light from the skin are measured with an internal spectrometer in the range 300-600 nm. SAF is the ratio between the average emitted light intensity per nm in the 420-600 nm range and the average reflected intensity per nm in the 300-420 nm range multiplied by 100.[5,6] Measurements were taken 10 cm below the elbow on the volar side of the forearm. There were no relevant differences in sex distribution, age or biochemical variables between those with and without SAF measurements performed.

Laboratory parameters

Blood samples were taken after an overnight fast and were placed at 4°C and transported from the Lifelines research site to the Lifelines laboratory, under tightly controlled and continuously monitored conditions. From the Lifelines laboratory, part of the samples were directly transferred to the central laboratory of the University Medical Center Groningen, to perform routine clinical chemistry assays. Hemoglobin concentration was measured using routine procedures on a XE2100-system (Sysmex, Japan). Glycated haemoglobin (EDTA-anticoagulated) was analyzed using a NGSP-certified turbidimetric inhibition immunoassay on a Cobas Integra 800 CTS analyser (Roche, the Netherlands). High-density lipoprotein cholesterol levels were measured using an enzymatic colorimetric method, triglycerides using a colorimetric UV method, both on a Roche Modular P chemistry analyzer (Roche, Switzerland). Serum creatinine was measured on a Roche Modular P chemistry analyzer (Roche, Switzerland) and renal function was calculated as estimated GFR with the formula developed by the Chronic Kidney Disease Epidemiology Collaboration.[7] High-sensitivity C-reactive protein (CRP) was measured with CardioPhase hsCRP (Siemens, Germany) and from 2012 with CRPL3 on a Roche Modular P chemistry analyzer (Roche, Switzerland). Serum vitamin B12 and folate concentration were measured by electrochemiluminescence immunoassay (Roche, Germany). Serum methylmalonic acid was measured by liquid chromatography-mass spectrometry (Spark Holland Symbiosis combined with Waters Quattro Premier XE). Serum iron, transferrin and ferritin were measured by ferrozine-based colorimetric assay, immunoturbidometric assay and electrochemiluminescence immunoassay respectively (Roche, Germany).

Definition of anemia

There is no worldwide accepted classification into subtypes of anemia. We subdivided individuals into three groups: anemia due to nutrient deficiency, anemia of inflammation and unexplained anemia. Iron deficiency was considered present when serum ferritin concentration <30 µg/L and transferrin saturation rate <16%. Transferrin saturation was calculated by dividing serum iron by total iron-binding capacity [transferrin (g/L) x 25]. Folate deficiency was defined as a serum folate level <9.8 nmol/L. Vitamin B12 deficiency was defined when vitamin B12 serum levels <145 pmol/L or serum methylmalonic acid concentration >340 nmol/L. When no evidence of a nutrient deficiency was established, other causes of anemia were evaluated. Anemia of inflammation was defined as present if the participant had (i) a CRP >5.0 mg/L or an absolute number of leukocytes >10 x 10^9/L or (ii) two or more of the following criteria: transferrin saturation rate <16%, serum ferritin concentration >100 µg/L or serum iron <10 µmol/L. Individuals with anemia that could not be classified into any of these previous categories were considered, by exclusion, to have unexplained anemia.[8,9]

SDC, Table 1. Baseline characteristics of anemic and non-anemic individuals

	**Anemic individuals (*n* = 3128)**	**Non-anemic individuals (*n* = 71475)**	**Data available (n)**	***P* value**
Male sex (%)	11.5	42.4	74603	<0.001
Age (years)	43.0 ± 11.2	44.1 ± 12.3	74603	<0.001
BMI (kg/m^2)	25.1 ± 4.5	26.0 ± 4.2	74588	<0.001
Hemoglobin (g/dL)			74603	
Males	12.5 ± 0.6	15.2 ± 0.9		<0.001
Females	11.3 ± 0.8	13.5 ± 0.8		<0.001
HbA1c (%)	5.6 ± 0.3	5.5 ± 0.3	74456	<0.001
HDL (mmol/L)	1.6 ± 0.4	1.5 ± 0.4	74602	<0.001
Triglycerides (mmol/L)	0.9 ± 0.5	1.2 ± 0.8	74603	<0.001
eGFR (ml/min/1.73m^2)	98.1 ± 15.3	96.9 ± 14.7	74603	<0.001
Current smokers (%)	13.4	21.6	74233	<0.001
Pack years (n)	0.0 (0.0 – 5.3)	0.5 (0.0 – 9.0)	71269	<0.001
Cups of coffee per day (n)	2.8 (1.3 – 4.6)	3.7 (1.9 – 4.6)	73380	<0.001

Data are given as mean ± SD, median (IQR) when not normally distributed, or percentage. BMI, body mass index; eGFR, estimated glomerular filtration rate; HbA1c, glycated hemoglobin; HDL, high-density lipoprotein; IQR, interquartile range; SD, standard deviation.

References

1 Scholtens S, Smidt N, Swertz MA, et al. Cohort Profile: LifeLines, a three-generation cohort study and biobank. *Int J Epidemiol* 2015; 44: 1172-1180.
2 Stolk RP, Rosmalen JG, Postma DS, et al. Universal risk factors for multifactorial diseases: LifeLines: a three-generation population-based study. *Eur J Epidemiol* 2008; 23: 67-74.
3 van der Ende MY, Hartman MH, Hagemeijer Y, et al. The LifeLines Cohort Study: Prevalence and treatment of cardiovascular disease and risk factors. *Int J Cardiol* 2017; 228: 495-500.
4 van Waateringe RP, Fokkens BT, Slagter SN, et al. Skin autofluorescence predicts incident type 2 diabetes, cardiovascular disease and mortality in the general population. *Diabetologia* 2019; 62: 269-280.
5 Koetsier M, Lutgers HL, de Jonge C, et al. Reference values of skin autofluorescence. *Diabetes Technol Ther* 2010; 12: 399-403.
6 Meerwaldt R, Graaff R, Oomen PH, et al. Simple non-invasive assessment of advanced glycation endproduct accumulation. *Diabetologia* 2004; 47: 1324-1330.
7 Levey AS, Stevens LA, Schmid CH, et al. A new equation to estimate glomerular filtration rate. *Ann Intern Med* 2009; 150: 604-612.
8 van Zeventer IA, de Graaf AO, Wouters HJCM, et al. Mutational spectrum and dynamics of clonal hematopoiesis in anemia of older individuals. *Blood* 2020; 135: 1161-1170.
9 Wouters HJ, van der Klauw MM, de Witte T, et al. Association of anemia with health-related quality of life and survival: a large population-based cohort study. *Haematologica* 2019; 104: 468-476.
